# Case report: Hydrometrocolpos conditioning recurrent urinary tract infections

**DOI:** 10.3389/fsurg.2022.869152

**Published:** 2022-10-11

**Authors:** Ewelina Malanowska, Mariola Krzyścin, Elzbieta Sowińska-Przepiera, Andrzej Starczewski, Tadeusz Sulikowski, Matteo Balzarro, Emanuele Rubilotta

**Affiliations:** ^1^ Department of Gynecology, Endocrinology and Gynecologic Oncology, Pomeranian Medical University, Szczecin, West Pomeranian, Poland; ^2^ Department of Endocrinology, Metabolic Diseases and Internal Medicine, Szczecin, West Pomeranian, Poland; ^3^ Department of General, Minimally Invasive, and Gastroenterological Surgery, Pomeranian Medical University in Szczecin, Poland; ^4^ Department of Urology, Azienda Ospedaliera Universitaria Integrata Verona, Verona, Italy

**Keywords:** hydrometrocolpos, recurrent urinary tract infections (rUTI), translabial ultrasound, vaginal septum, adolescent gynecology

## Abstract

We present a case of a 12.5-year-old girl who has suffered from recurrent urinary tract infections for many years but has never undergone a detailed diagnostic process. Only as a teenager did she complain of acute pain in her lower abdomen and it turned out that her genital organs had not properly developed. She had an obstructive defect in the reproductive tract. When there was a significant amount of discharge collected in the lumen of the genital tract and the organs had distended, acute pain appeared, which allowed us to make the diagnosis. In the diagnostic process, transperineal ultrasonography turned out to be extremely helpful, allowing us to establish the type and thickness of the obstruction. The patient underwent excision of transverse vaginal septum, and postoperative silicon dilators were used to prevent the recurrence of the obstruction. There was no recurrence of urinary infection or complications during the 11 months of follow-up.

## Introduction

Congenital agenesis of the lower vagina is a rare condition that may be a predisposing factor for recurrent urinary tract infection (rUTI). This disorder is caused by a blockage of the outflow and the subsequent accumulation of fluids in the vagina and uterus. The three types of female genital obstruction are hymen atresia, transverse vaginal septum, and partial vaginal atresia. Diagnosis can be made late in pregnancy (at around 30 weeks of gestation) or early in the neonatal period with hydrometrocolpos (HMC). HMC is defined as the distension of the uterus and the vagina caused by fluid other than blood or pus. However, the obstruction in the vagina is much more commonly associated with accumulation of blood, which is known as hematocolpometra (HCM). It is typically diagnosed in adolescence and only very rarely during adulthood, with symptoms of primary amenorrhea, cyclic abdominal pain, and sometimes an abdominal mass (1). Recurrent UTI can be a misunderstood symptom of this pathological condition.

Because HMC is a very rare disorder, it is not even considered in the diagnostic process of rUTI in young girls. Girls suffering from rUTI should be thoroughly diagnosed not only by a urologist but also by a gynecologist, due to the possibility of hidden defects of vaginal patency. When clinical symptoms occur, failure to undertake prompt and proper therapy may result in kidney damage (2).

Our study aims to present a case of transverse vaginal septum that was recognized only in adolescence in a girl who had previously suffered from rUTI for many years.

## Case presentation

A 12.5-year-old girl presented with abdominal pain polyuria and hematuria. She claimed to have similar ailments as those admitted about a year ago. She was then referred to a urologist, who had suspected an imperforate hymen. An attempt was made to puncture a recognized vaginal obstruction and only 10 ml of transparent fluid was drained. Finally, she was diagnosed with cystitis at that time and was given an antibiotic treatment that relieved the symptoms within 2 days.

**Figure 1:**
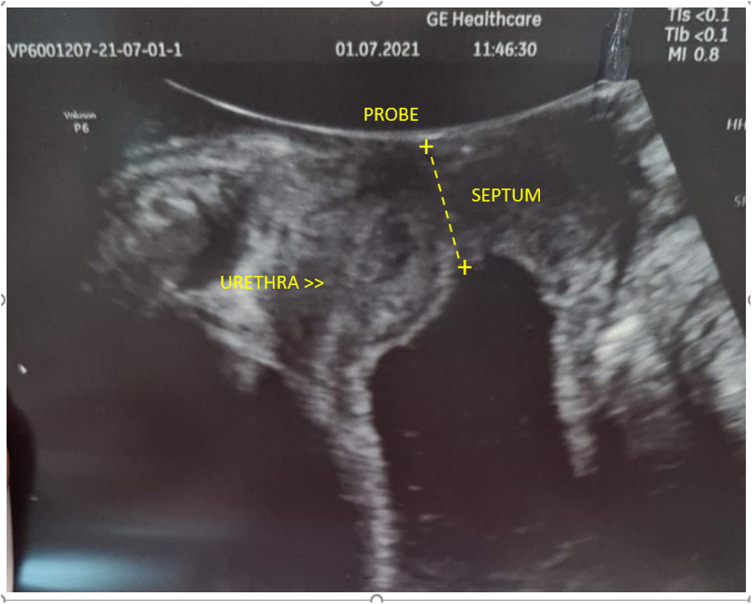
Transperineal ultrasound pictures: transverse vaginal septum approximately 1.2 cm in thickness appears as a hyperechoic structure. Below, it is seen as a large hypoechoic mass that responds to the accumulation of fluids in the vagina.

The patient was admitted to our tertiary care center for further evaluation. In her clinical history, she had suffered from rUTIs with different bacteria documented in each episode for many years. Last year, she was treated five times with antibacterial and chemotherapeutic agents due to UTI. Physical examination on admission revealed Tanner stage III breast and genital development. A tense lower-abdominal mass rose to a level of 10 cm above the pubic symphysis. A perineal inspection revealed normally developed labia majora, labia minora, and clitoris. The hymeneal ring was readily identified, and the vaginal pit was very shallow with no bulging at the outlet. By ultrasound, both kidneys were within the normal limits for age, without any signs of urinary retention. Longitudinal and transverse transabdominal sonographic scanning demonstrated a large, hourglass-shaped, hypoechoic abdomen-pelvic mass. A transperineal ultrasound (US) was performed using a 3.5-mHz convex transducer (GE Voluson E6), clearly demonstrating the septum approximately 1.2 cm in thickness appearing as a hyperechoic shelf with the large hypoechoic mass above (Figure 1). Urinalysis revealed a recurring UTI. Vaginoscopy was attempted, but it was not possible as there was an obstruction. Magnetic resonance imaging (MRI) was not performed because there was no such possibility in the emergency mode.

### Surgical management

**Figure 2:**
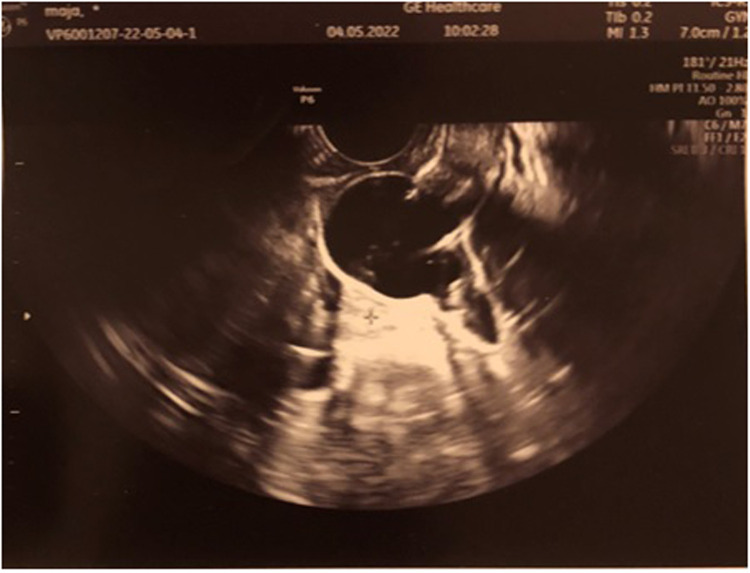
Intraoperative transrectal sonography presents hematocolpos.

**Figure 3:**
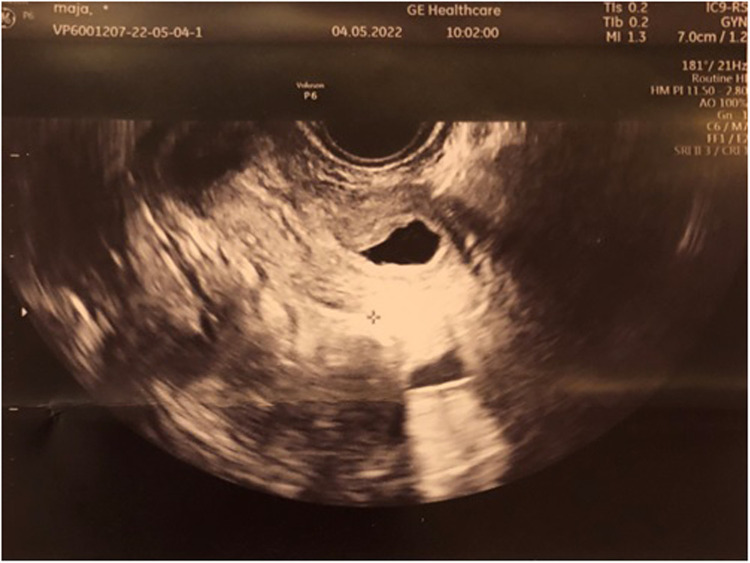
Intraoperative transvaginal sonography shows the resolution of hematocolpometra.

We set the diagnosis of UTI accompanied by HCM resulting from a low-transverse vaginal septum and qualified the patient for operative vaginal septum resection. Intraoperative transrectal sonography was performed (Figure 2). During surgery, while opening the vaginal wall, 150 ml of hemolyzed discharge was drained from the vagina. A 2-cm thick low-transverse septum was excised. Decompression of the HCM yielded a thicker septum, as estimated with the US on admission. The superior vaginal canal measured approximately 5 cm long and was lined with normal-appearing epithelium. The circumferential margins of the inferior and superior vaginal canals were carefully mobilized and anastomosed over the defect. Figure 3 depicts the resolution of HCM in transvaginal intraoperative sonography (sagittal view). In the speculum, an intact cervix was visualized. Subsequently, an intraoperative cystoscopy was performed to exclude vesicovaginal or uterine fistula, but no abnormalities were seen.

### Follow-up

The postoperative vaginal examination allowed digital palpation without appreciable vaginal stenosis. Because the risk of vaginal stenosis was high, the girl was advised to use a vaginal dilator at night to prevent constriction after reconstruction. The patient and her mother were guided to smear a silicone vaginal dilator with neutral gel and gently insert into the vagina initially every day, then every few days overnight. During menstruation, the use of vaginal tampons was recommended. The patient received an antibiotic, and 1 week after the procedure, the patient's urine was tested. Painless, spontaneous monthly menses followed the surgical repair, and there was no UTI observed in the 12th month after the procedure.

## Discussion

In children, in the case of rUTI, an underlying disorder should be meticulously researched because the lack of diagnosis of the predisposing factor for rUTI may expose the patient to chronic infections, antibiotic therapy failure, and eventually lead to kidney damage (3). It is estimated that approximately 8.4% of girls and 1.7% of boys under the age of six suffer from UTI within the first 6 years of life (2). Approximately 30% of this group will experience a relapse within the next 6–12 months. Furthermore, rUTI may occur without the typical signs and symptoms, as in the case that they are sustained by genitourinary anomalies. HMC can be a challenging diagnosis in young girls, especially before menarche, and when there is no reported in the patients without a large amount of discharge accumulated in the closed vagina. Although there might be some symptoms in neonates or babies, such as abdominal distention that can lead the physician to think of an obstruction, this anomaly often remains unrecognized until puberty. For this reason, this condition is often not taken into consideration in the diagnostic process (4). Still, gathering the fluid in the vagina without being drained can make it contaminated. The pressure change between the closed vagina and the urinary tract may activate the micro fistulas above the level of obstruction, and trigger the symptoms.

An accurate history, a physical examination, and a transperineal ultrasound seem to be crucial to set the proper diagnosis in case of genital tract obstruction. Because we did not have access to MRI, the transperineal ultrasound scan was pivotal in setting the diagnosis and determining the cause of HMC in our patient. Although having some limitations, especially in obstructed anomalies such as underestimating the length of the vaginal canal, the transperineal technique of scanning is fast, cheap, and accurate. It allows for the estimation of the thickness and level of the vaginal pathologies that are obscured behind the hymen, especially in virgin females. Finally, it may be very helpful in planning the therapy (5). In this case, despite not having visualized but being aware of possible connections between the closed vagina and the bladder or urethra, we decided to perform also intraoperative cystoscopy.

First, the patient was treated by urologists in her childhood because the rUTI was the only sign of abnormality. During the diagnostic process of rUTI in girls, urologists may be more focused on urinary tract abnormalities and less on congenital anomalies that concern the reproductive tract alone. Interdisciplinary cooperation should be taken into consideration to determine the treatment options (1). Because the patient began menstruating and the other symptoms appeared, the diagnosis became much easier.

Clinically, the various types of vaginal blockage present similarly; however, differentiation is essential for appropriate surgical planning (6, 7). The approaches to labia separation, imperforate hymen, transverse vaginal septum, and vaginal agenesis are completely different (8, 9). Vulval synechia requires local estrogen therapy, a simple incision in the midline, or both. In the case of HMC caused by imperforate hymen, a bulging membrane should be visualized in the vulval cleft. Therefore, the simple membrane incision is the definitive treatment, which does not require the use of dilators (6). A transverse vaginal septum can be placed at three different levels: proximal, medial, and distal parts of the vagina. The thickness of the septum can also vary from very thin to very thick, but both sides of the septation are covered with vaginal mucosa. It is imperative to excise the septum and anastomose on both edges of the cut mucosa in order to create a continuity of the vaginal canal. In thick septations, a vaginal mobilization can be necessary (10). A simple incision and drainage of the accumulated fluid is not sufficient. When doing this, the incision scarifies quickly, and a rapid symptom recurrence is usually observed. Vaginal atresia is characterized by a lack of vaginal entrance at the level of perineum. The proximal part of the vagina can vary in length. In such cases, vaginal mobilization from the perineum or abdomen side can be planned. In certain cases, replacement of the vagina is required (7). In both transverse septation and vaginal atresia, a subsequent vaginal dilatation is highly recommended.

There are several surgical approaches for removing vaginal septum described, starting from classical excision with a scalpel and scissors, after which the edges are sutured for hemostasis. Then there's electroresection, which is probably used most frequently nowadays. Finally, there are many innovative methods using different instruments described such as laparoscopic long bipolar forceps, harmonic scalpel, LigaSure (Medtronic Inc., Doral, FL), GIA, and Endo GIA device (11–14). In critical cases, with the development of acute kidney failure, bladder injury, urinary leakage into the abdominal cavity, or other abdominal complications, laparotomy is recommended (15).

## Conclusion

Obstacles to patency of genital tract should be considered in young females suffering from rUTI. Adequate and quick proceeding in HMC is necessary to avoid further complications. Successful management of rUTI requires collaboration between urologists and gynecologists.

## Data Availability

The original contributions presented in the study are included in the article, further inquiries can be directed to the corresponding author.
